# CD44-Targeted Lipid Polymer Hybrid Nanoparticles Enhance Anti-Breast Cancer Effect of *Cordyceps militaris* Extracts

**DOI:** 10.3390/pharmaceutics15061771

**Published:** 2023-06-20

**Authors:** Jiraphong Suksiriworapong, Nutthachai Pongprasert, Somnuk Bunsupa, Vincenzo Taresco, Valentina Cuzzucoli Crucitti, Thitapa Janurai, Pornpoj Phruttiwanichakun, Krisada Sakchaisri, Amaraporn Wongrakpanich

**Affiliations:** 1 Department of Pharmacy, Faculty of Pharmacy, Mahidol University, Bangkok 10400, Thailand; 2 Division of Postharvest Technology, School of Bioresources and Technology, King Mongkut’s University of Technology Thonburi, Bangkok 10150, Thailand; 3 Department of Pharmacognosy, Faculty of Pharmacy, Mahidol University, Bangkok 10400, Thailand; 4 School of Chemistry, University of Nottingham, Nottingham NG7 2RD, UK; 5 Centre for Additive Manufacturing and Department of Chemical and Environmental Engineering, University of Nottingham, Nottingham NG7 2RD, UK; 6 Department of Pharmacology, Faculty of Pharmacy, Mahidol University, Bangkok 10400, Thailand

**Keywords:** *Cordyceps militaris*, anticancer, hyaluronic acid, lipid polymer hybrid nanoparticles, breast cancer, targeted nanoparticles, CD44 receptor, poly (glycerol adipate)

## Abstract

This study aimed to improve the anticancer effect of *Cordyceps militaris* herbal extract (CME) on breast cancer cells with hyaluronic acid (HYA) surface-decorated lipid polymer hybrid nanoparticles (LPNPs) and evaluate the applicability of a synthesized poly(glycerol adipate) (PGA) polymer for LPNP preparation. Firstly, cholesterol- and vitamin E-grafted PGA polymers (PGA-CH and PGA-VE, respectively) were fabricated, with and without maleimide-ended polyethylene glycol. Subsequently, CME, which contained an active cordycepin equaling 9.89% of its weight, was encapsulated in the LPNPs. The results revealed that the synthesized polymers could be used to prepare CME-loaded LPNPs. The LPNP formulations containing Mal-PEG were decorated with cysteine-grafted HYA via thiol-maleimide reactions. The HYA-decorated PGA-based LPNPs substantially enhanced the anticancer effect of CME against MDA-MB-231 and MCF-7 breast cancer cells by enhancing cellular uptake through CD44 receptor-mediated endocytosis. This study demonstrated the successful targeted delivery of CME to the CD44 receptors of tumor cells by HYA-conjugated PGA-based LPNPs and the new application of synthesized PGA-CH- and PGA-VE-based polymers in LPNP preparation. The developed LPNPs showed promising potential for the targeted delivery of herbal extracts for cancer treatment and clear potential for translation in in vivo experiments.

## 1. Introduction

The fruiting body of *Cordyceps militaris* (CM) has gained profound importance as a source of natural products with diverse biological activities and has been extensively used as a crude drug and a functional food in Asia [1,2]. Several pharmacological activities of CM have been reported, such as antimicrobial, antifungal, antidiabetic, anti-inflammatory, antiaging, and neuroprotective activities [2,3,4,5]. CM contains a large variety of bioactive constituents, including cordycepin, ergosterol, carotenoids, essential amino acids, carotenoids, minerals, vitamins, nucleosides, and sterols [1]. Apart from these activities, the CM extract (CME) from the fruiting body exerts an anticancer activity against numerous cancer cells, such as ovarian, breast, lung, colon, and skin cancers [6,7,8,9,10,11]. The main active constituent of CME, that possesses anticancer activity, is cordycepin, an adenosine nucleoside analog [1]. Although cordycepin exhibits an interesting in vitro anticancer activity, the limited use of cordycepin in clinical states has been attributed to the lack of specificity to tumors, short plasma half-life, and rapid clearance from the blood circulation, resulting in low accumulation at the spatial site of action [12,13].

Nanotechnology, particularly nanoparticles, has emerged as a promising solution for the precise delivery of several natural bioactive compounds to enhance therapeutic activities [14,15,16]. In recent decades, numerous studies have demonstrated the role of nanoparticles in increasing drug accumulation at the tumor site and regulating the release of active compounds to extend administration periods [17,18]. However, delivery of the extract is still challenging due to the possibility of the complex interactions of various constituents with formulation excipients and blood components, the variation in bioactive components, and the complexity of biological systems, including dissolution in biological fluids, absorption, metabolism, and bioavailability of phytochemical components [19,20]. Targeted nanoparticles govern active targeting, which involves specific receptors on the surface of the cell and ligand-receptor interactions, to deliver bioactive compounds precisely and accurately to the cells. CD44 is a cell surface molecule; it plays a significant role in cell proliferation, cell differentiation, cell migration, and angiogenesis in many cancer cells. This receptor is recognized as a potential target in cancer treatment due to its high expression in many cancer cells compared with that in normal cells [21]. Hyaluronic acid (HYA) is well known as a CD44-targeting ligand and has frequently been used to decorate the nanoparticle surface for delivery to CD44-overexpressed cancer cells owing to its specificity toward CD44 receptors [22,23,24,25].

A few previous studies have established delivery systems for active cordycepin, namely poly(lactide-*co*-glycolide) (PLGA) nanoparticles, liposomes, and bilo/niosomes [26,27,28]. Nevertheless, the delivery of CME containing cordycepin is challenging as it contains various bioactive constituents. Only a few studies have reported the delivery of cordycepin-containing extracts using nanoemulsions [29], liposomes [30], and gelatin nanoparticles [31]. Although the CME-loaded gelatin nanoparticles showed a toxicity toward A549 lung cancer cells that was comparable to the extract alone, the system could significantly inhibit the migration of the cells. Until now, there have been no reports on the successful enhancement of the anticancer activity of CME using targeted nanoparticles. The CD44-targeted nanoparticles containing CME may enhance the anticancer effect of the extract and selectively target CD44 receptor-overexpressed cancer cells.

Breast cancer is the most common cause of cancer death in women worldwide. Chemotherapy is the treatment of choice for breast cancer; however, it remains insufficient due to rapid clearance from the body and a lack of selectivity and specificity toward the targeted tumor tissues [32]. The CD44 receptor expression level in breast cancers rises proportionally to the grade and stages of invasiveness [33,34]. Triple-negative MDA-MB-231 and HER-negative MCF-7 cells show variable degrees of invasiveness and may express different levels of CD44 receptors. Therefore, this study aimed to improve the anticancer effect of CME on breast cancer cells by CD44-targeted nanoparticles. The HYA-conjugated lipid polymer hybrid nanoparticles (LPNPs) were developed using poly(glycerol adipate) (PGA) as a polymeric material and phosphatidylcholine (PC) as a lipid component. LPNPs have recently been introduced as a new generation of delivery systems to utilize the unique characteristics of liposomes and polymeric nanoparticles in order to overcome their individual drawbacks, such as structural disintegration, limited circulation time, and component leakage [35,36]. LPNPs are composed of a polymeric core, a lipid layer surrounding the core, and an outer steric stabilizing PEG-based material that prevents destruction by immune systems. The second objective of this study was to evaluate the feasibility of poly(glycerol adipate) (PGA) for LPNP preparation. Previous studies demonstrated that the incorporation of lipophilic cholesterol (CH) and vitamin E (VE) in the formulations enhanced the stability of lipid-based nanoparticles and may exert anticancer activity against selected cancer cell lines [37,38]. Thus, PGA modifications were performed by incorporating CH and VE along the polymer structure [39]. The grafting of PGA with CH and VE may result in polymer–lipid hybrid characteristics, which may facilitate the formation of LPNPs and the entrapment of CME. The CH- and VE-grafted PGA polymers were also PEGylated with maleimide-ended polyethylene glycol (Mal-PEG) to provide steric stabilization by the PEG chain and to functionalize with HYA-cys at the terminal maleimide group. A mixture of Mal-PEG-free and Mal-PEG-derivatized PGAs was utilized in the formulations. The anticancer activity of CME-loaded LPNPs in comparison with that of CME was evaluated in triple-negative MDA-MB-231 and HER-negative MCF-7 breast cancer cells. Eventually, the cellular uptake of HYA surface-decorated LPNPs was also assessed in both cell lines using coumarin-6 as a fluorescence probe.

## 2. Materials and Methods

### 2.1. Materials

PGA was synthesized as previously described [40]. Thiolation of HYA was performed according to the published method [41] by the conjugation of HYA with cys using EDC/NHS as coupling agents (see Supporting Information for details).

Cordycepin (Wuhan ChemFaces Biochemical Co., Ltd., Wuhan, China); 1-ethyl-3-(3-dimethylaminopropyl)carbodiimide hydrochloride (EDC, Fluka, Tokyo, Japan); HYA (sodium salt, MW 8000–15,000 g/mol, Biosynth Limited, Compton, UK); maleimide poly(ethylene glycol) acetic acid (Mal-PEG, MW 2000 g/mol, Jenkem Technology Co., Ltd., Beijing, China); PC (Lipoid S100, Lipoid GmbH, Ludwigshafen, Germany); PLGA (50:50, inherent viscosity 0.59 dL/g, MW 53.4 kDa, Durect Corporation, Birmingham, AL, USA); and polyvinyl alcohol (PVA, Mowiol^®^ 8–88, MW 67 kDa, Sigma-Aldrich Pte. Ltd., Singapore) were employed as received. Cholesteryl hemisuccinate (CH); 4-(dimethylamino)pyridine (DMAP); *N*-hydroxysuccinimide; and tocopheryl succinate (VE) were obtained from Tokyo Chemical Industry Co., Ltd. (Kita-ku, Tokyo). Coumarin-6; L-cysteine hydrochloride (cys); *N*,*N*’-dicyclohexylcarbodiimide (DCC); and 5,5′-dithiobis(2-nitrobenzoic acid) were purchased from Sigma-Aldrich, Saint Louis, MO, USA. Dulbecco’s modified eagle medium (DMEM, high glucose, pyruvate, Invitrogen™, Thermo Fisher Scientific Inc., Waltham, MA, USA); Hoechst 33,342 (Invitrogen™, Thermo Fisher Scientific Inc., Waltham, MA, USA); fetal bovine serum (FBS, Life Technologies Corporation, Eugene, OR, USA); and thiazolyl blue tetrazolium bromide (MTT, AppliChem GmbH, Damstadt, Germany) were of cell culture or molecular grade.

MDA-MB-231 (ATCC number HTB–26^TM^) and MCF-7 (ATCC number HTB–22^TM^) breast cancer cell lines were attained from American Type Culture Collection, Manassas, VA, USA.

### 2.2. Cultivation and Extraction of CM Fruiting Body

The culture process was conducted at the School of Bioresources and Technology, King Mongkut’s University of Technology Thonburi, Bangkok, Thailand. *C. militaris* seeds were prepared by inoculating the fungus mycelia; and then they were cultured statically in sterile liquid culture medium (containing boiled and mashed potato (20% *w*/*v*), glucose (2% *w*/*v*), peptone (0.2% *w*/*v*), yeast extract (0.2% *w*/*v*), MgSO_4_ (0.1% *w*/*v*), and vitamin B1 (0.003% *w*/*v*) in distilled water) for 24 h, followed by shaking in the dark at 150 rpm, 22 °C for 5 days. Once the mycelia covered the full medium surfaces, they were then cultured under white LED lights (1000 lux) for primordia induction and fruiting body growth. The dark-grown mycelia were inoculated on a sterile brown rice-based solid medium, consisting of 1000 g of Hom Mali cultivar brown rice, 20 g of glucose, 7 g of yeast extract, 5 g of peptone, 1.5 g of vitamin B1, 0.5 g of MgSO_4_, 2 eggs, and 1 L of water, in the dark for 5 days until the mycelia covered the full medium surface. For primordia induction, the culture bottles were illuminated under different light combinations (12 h light and 12 h darkness, 22 °C, 80% humidity). After 70 days, the fruiting bodies with a length of 8 cm or more were harvested, dried, and pulverized.

The fruiting body powder (1 g) was extracted with 50% *v/v* ethanol (20 mL) using the ultrasonic-assisted extraction method according to the reported method [42], with minor modifications. The wet mass was sonicated by an ultrasonic bath (GT Sonic-D6, GuangDong GT Ultrasonic Co., Ltd., Shenzhen, China) at 40 kHz, 60 °C for 60 min. The sample was filtered through Whatman No. 1 filter paper. The filtrate was collected, and the extraction of residual mass was repeated twice. The filtrate was combined, and the solvent was evaporated using a rotary evaporator at 45 °C. The crude extract was further dried using the Christ Alpha 1–4 lyophilizer (Martin Christ Gefriertrocknungsanlagen GmbH, Göttingen, Germany) for 48 h. The dried extract was kept in a desiccator and light-protected until further use.

### 2.3. Analysis of CM Extract (CME)

The content of cordycepin in the samples was standardized by a high-performance liquid chromatography (HPLC) apparatus (Prominence, Shimadzu Corporation, Kyoto, Japan) according to the reported method [43], with some modifications. The sample was eluted through a reverse phase C18 Gemini-NX column (150 × 4.6 mm, 5 µm, Phenomenex Inc., Torrance, CA, USA) with a C18 guard column, at 35 °C. A mixture of 10% *v/v* methanol in water was used as a mobile phase at a flow rate of 1 mL/min. Cordycepin was detected at a wavelength of 260 nm. The cordycepin content in the sample was analyzed over the standard concentration range of 0.1–100 µg/mL. The HPLC chromatograms of cordycepin and CME are provided in Figure S2.

### 2.4. Modification of PGA

The modification of PGA was performed by grafting with CH and VE using a carbodiimide coupling reaction. The molar ratio of the glycerol adipate repeating unit to CH or VE was 1.0:0.5. PGA-CH was synthesized as previously reported [15]. In the case of PGA-VE, PGA (equivalent to 3.71 mmol of hydroxyl group) was dissolved in anhydrous THF (40 mL), followed by sequential additions of DMAP (1.11 mmol), VE succinate (4.45 mmol), and DCC (4.45 mmol). The reaction was stirred under an inert gas at room temperature for 24 h. The precipitate was removed by centrifugation at 4500 rpm for 15 min, and the solvent was evaporated by a rotary evaporator. The crude polymer was sequentially washed with 0.2 M NaOH, 0.2 M HCl, and deionized water. The polymer was further purified by dialysis (MWCO 6–8 kDa) against methanol for 24 h. Finally, the polymer was dried in a glass vacuum oven at 45 °C. The obtained PGA-CH and PGA-VE were subsequently conjugated with Mal-PEG. Briefly, Mal-PEG (0.262 mmol) was activated with DCC (0.314 mmol) in anhydrous THF under nitrogen gas for 2 h. Afterwards, the solution of PGA-CH or PGA-VE (0.192 mmol of hydroxyl group) and DMAP (0.230 mmol) was added to the previous reaction mixture. The reaction was conducted in a nitrogen atmosphere for 72 h. The removal of precipitates by centrifugation at 4500 rpm for 15 min and the solvent evaporation by a rotary evaporator were performed. The polymer was further purified by dialysis (MWCO 12 kDa) against methanol for 24 h. Then, methanol was removed under reduced pressure, and the polymer was dried in a vacuum oven at 45 °C for 14 h.

### 2.5. Characterization of Polymers

The synthesized polymers were characterized by proton nuclear magnetic resonance spectroscopy (^1^H NMR, Bruker Avance NMR machine, Bruker Corporation, Rheinstetten, Germany), attenuated total reflectance infrared spectroscopy (ATR-IR, Nicolet iS5 FTIR Spectrometer, Thermo Fisher Inc., Waltham, MA, USA), and gel permeation chromatography (GPC, Agilent 1260 infinity GPC apparatus, Agilent Technologies, Inc., Cheadle, UK). ^1^H NMR was performed at 300 MHz and 25 °C in acetone-*d_6_*. ATR-IR spectroscopy was conducted over the range of 4000–500 cm^−1^ at a resolution of 4 cm^−1^. GPC was used to measure the number-average molecular weight (M_n_) and molecular weight distribution (M_w_/M_n_). The sample was eluted through two mixed C columns using THF as a mobile phase at 1 mL/min, 30 °C. The peak of the eluted polymer was detected by a light scattering and refractive index multi-detector. The M_n_ and M_w_/M_n_ were computed from the standard polymethylmethacrylate (PMMA) calibration curve.

### 2.6. Preparation of LPNPs

Generally, the PGA-CH- and PGA-VE-based LPNPs for encapsulation of CME were prepared by a water-in-oil-in-water (w/o/w) double emulsion-solvent evaporation technique using probe sonication [44]. In an organic phase, PC was used as a lipid component, whereas PLGA, PGA-CH, PGA-VE, and their Mal-PEG derivatives were employed as polymer components. The weight ratio of the lipids and polymers was predetermined as 25:75% *w*/*w*. Briefly, CME (2.5 mg) was dissolved in 0.253 mL of water phase 1 (W1), containing 20% *v/v* ethanol, 10 mM HCl, and 0.2% *w/v* HYA. The solution was shaken at 200 rpm at room temperature overnight to allow the physical adsorption of CME and HYA. For blank LPNPs, W1 without CME was used to prepare the LPNPs. W1 was then emulsified in 1.0 mL of CH_2_Cl_2_, containing 25 mg of lipids and polymers, using an ultrasonic processor with a timer and pulser (Sonics & Materials, Inc., Newtown, CT, USA) at 40% amplitude for 30 s. For the coumarin-6-labeled LPNPs, coumarin-6 was dissolved together with the lipids and polymers in the organic phase prior to emulsification. The primary emulsion was immediately emulsified in 2.5 mL of water phase 2 (W2), consisting of 1% PVA at 40% amplitude continuously for 120 s, followed by pulse sonication for 30 s for six cycles. The emulsion was stirred for 2 h to evaporate DCM. The LPNPs were then collected.

HYA was conjugated on the surface of the LPNPs through a thiol-maleimide reaction, as reported elsewhere [45,46], with some modifications. The formulations containing Mal-PEG derivatives were used in this study. The Mal pendants of the LPNPs reacted with HYA-cys at a 15:1 mole ratio of Mal to thiol groups. The LPNPs (1.8 mL) were mixed with 0.5 mL of HYA-cys in phosphate buffer solution, pH 7.0, flushed with nitrogen gas, and shaken in the dark at 200 rpm, 30 °C, for 1.5 h. Subsequently, the HYA-conjugated LPNPs (HYA/50PEG/CH and HYA/50PEG/VE LPNPs) were collected and used without further purification.

### 2.7. Characterization of LPNPs

The measurement of particle size, polydispersity index (PDI), and zeta potential (ZP) was performed by Zetasizer NanoZS (Malvern Instrument Ltd., Malvern, UK). A He-Ne laser at a wavelength of 633 nm was used for the measurement, at an angle of 173° and 25 °C. The sample (5-fold dilution in water) was measured in triplicate. The morphology of the LPNPs was observed by transmission electron microscope (TEM, JEOL JEM-1400, JEOL Ltd., Tokyo, Japan). The sample was stained with 1% uranyl acetate on a carbon-coated copper grid. The yield of LPNPs was evaluated after drying the LPNPs in a hot air oven at 45 °C until a constant weight of the sample was obtained, and it was calculated based on the theoretical solid content of the formulation.

The drug loading (%DL) and entrapment efficiency (%EE) were calculated according to Equations (1) and (2), respectively. The total amount of CME content in the LPNPs was analyzed by HPLC. The CME was extracted from the dried LPNPs by first dissolving in 0.1 mL of acetone with a subsequent sonication for 15 min. Then, 0.45 mL of methanol and 0.45 mL of water were sequentially added, and the sample was sonicated for another 15 min. The precipitate was centrifuged at 13,000 rpm for 30 min. The supernatant was collected and analyzed for the total amount of CME content by HPLC. The unencapsulated CME was separated from the LPNPs by ultrafiltration using a centrifugal filter device (MWCO 3 kDa, Amicon^®^ Ultra, Merck KGaA, Darmstadt, Germany) and centrifugation at 13,000 rpm for 30 min. The amount of unencapsulated CME in the filtrate was analyzed by HPLC.

(1) $$\%\mathrm{DL}=\frac{\mathrm{Total}\;\mathrm{amount}\;\mathrm{of}\;\mathrm{CME}-\mathrm{Amount}\;\mathrm{of}\;\mathrm{Unencapsulated}\;\mathrm{CME}}{\mathrm{Solid}\;\mathrm{content}\;\mathrm{of}\;\mathrm{LPNPs}}\times 100$$

(2) $$\%\mathrm{EE}=\frac{\mathrm{Total}\;\mathrm{amount}\;\mathrm{of}\;\mathrm{CME}-\mathrm{Amount}\;\mathrm{of}\;\mathrm{Unencapsulated}\;\mathrm{CME}}{\mathrm{Amount}\;\mathrm{of}\;\mathrm{CME}\;\mathrm{initially}\;\mathrm{added}}\times 100$$

An amount of the free maleimide group on the surface of the unconjugated LPNPs was measured by Ellman’s assay, as previously reported [47,48]. In addition, the HYA-conjugated LPNPs were centrifuged using a centrifugal filter (MWCO 30 kDa, Amicon^®^ Ultra, Merck KGaA, Darmstadt, Germany) at 13,000 rpm for 30 min. The filtrate was collected for analysis of the unbound HYA-cys by Ellman’s assay.

### 2.8. Release Study

The release of CME from the HYA/CME-50PEG/CH and HYA/CME-50PEG/VE LPNPs was investigated in isotonic phosphate buffered saline, pH 7.4 (iPBS) using the dialysis method [16,49]. The release of the CME solution was also evaluated for comparison. In brief, 1 mL of samples was added to a dialysis bag (MWCO 3.5 kDa, Spectra/Por^®^, Spectrum Laboratories, Inc., Rancho Dominguez, CA, USA). After being tightly sealed, the sample was immersed in 10 mL of 0.03 M iPBS and shaken at 100 rpm, 37 °C using an incubator shaker. At predetermined time intervals, 1 mL of release medium was taken and immediately replenished with an equal volume of fresh release medium. The sample was then analyzed by HPLC.

### 2.9. Cytotoxicity Study

The toxicity of the CME-loaded LPNPs was evaluated in MDA-MB-231 and MCF-7 breast cancer cells using an MTT assay. The MDA-MB-231 and MCF-7 cells were cultured in DMEM supplemented with 10% FBS and 1% penicillin/streptomycin. Both cells were seeded in a 96-well plate at a density of 5 × 10^3^ cells/well and incubated in a 5% CO_2_ humidified incubator at 37 °C for 24 h. The old medium was replaced with various concentrations of CME-loaded LPNPs dispersed in the culture medium. The cells were further incubated for 24, 48, and 72 h. Afterwards, the MTT solution was added and incubated with the cells for another 2 h. The medium was discarded, and DMSO was added to solubilize the formazan products. After incubating for 15 min, the absorbance was measured by a microplate reader (CLARIOstar, BMG LABTECH^®^, Ortenberg, Germany) at a wavelength of 570 nm. The cell viability was computed, and the IC_50_ values were calculated using GraphPad^®^ Prism 7 software (GraphPad Software Inc., Boston, MA, USA).

### 2.10. Cellular Uptake Study

The uptake of coumarin-6-labeled LPNPs was investigated in MDA-MB-231 and MCF-7 breast cancer cells using flow cytometry. The cells were seeded in a 12-well plate at a density of 3 × 10^5^ cells/well. After 24 h of incubation in an incubator at 37 °C, coumarin-6-labeled LPNPs in FBS-free culture medium at 62.5, 125, and 250 µg/mL were added and incubated with the cells for 1, 2, and 4 h. Subsequently, the cells were washed twice with PBS, trypsinized with 0.25% trypsin/EDTA, and quenched with serum-containing media. The cells were removed from the plate, centrifuged, washed with PBS, and finally fixed with 4% paraformaldehyde. For quantitation of the uptake efficiency, the fluorescence intensity was measured by a flow cytometer (BD FACSVerse^TM^, BD Biosciences, Franklin Lakes, NJ, USA). The uptake efficiency ratio was calculated based on the measured mean fluorescence intensity per event relative to the initial fluorescence intensity of the LPNPs. The experiments were performed in triplicate. For qualitative measurement, the cellular uptake of the LPNPs was visualized by the EVOS M7000 imaging system (Thermo Fisher Scientific Inc., Waltham, MA, USA). To visualize the nuclei in the fixed cells, Hoechst 33,342 staining was performed for 30 min, and the cells were fixed again with 4% paraformaldehyde prior to visualization.

### 2.11. Statistical Analysis

The data are expressed as mean ± S.D. For paired and multiple comparisons, the t-test and one-way ANOVA with the Scheffe test applied post hoc were used, respectively. The GraphPad^®^ Prism 7 software was employed for all analyses. Differences were considered statistically significant at a *p*-value of 0.05 or less.

## 3. Results and Discussion

### 3.1. Extraction of CM Fruiting Bodies

In the preliminary study, an ultrasonic-assisted technique was compared with a conventional technique for the extraction of CM (Table S1). The ultrasonic-assisted method gave a higher CME yield than the conventional method. Meanwhile, both methods yielded comparable contents of cordycepin and adenosine. The extraction period needed for the ultrasonic-assisted technique consumed 24 times shorter, and a five-fold lower volume of solvent was used. Therefore, it was chosen for the extraction of CM. By ultrasonic-assisted extraction, the CME yield was 9.89% *w*/*w*, based on the dry weight of the powdered fruiting bodies. The CME was standardized for its cordycepin and adenosine contents using HPLC. The contents of cordycepin and adenosine in the extract were found to be 10.55 ± 0.01% *w*/*w* and 0.046 ± 0.002% *w*/*w*, respectively. In the previous reports, it was noted that the extract from *C. militaris* contained not only cordycepin and adenosine but also other bioactive compounds, such as uridine, inosine, β-glucan, ergosterol and its derivatives, ergothioneine, γ-butyric acid, lovastatin, and so on [2,50,51,52]. These may potentiate the effect of cordycepin in killing cancer cells.

### 3.2. Modification of PGA Polymers

**Figure 1:**
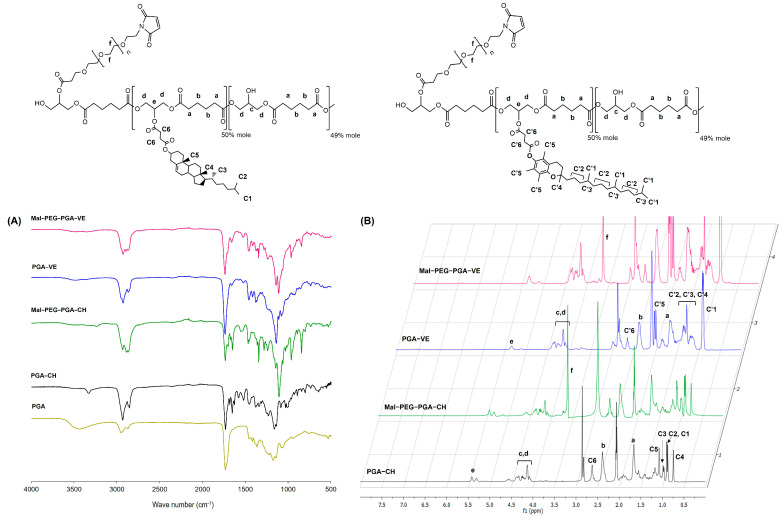
ATR-IR (**A**) and ^1^H NMR (**B**) spectra of PGA-CH, Mal-PEG-PGA-CH, PGA-VE, and Mal-PEG-PGA-VE polymers.

PGA has been successfully modified with lipophilic compounds and utilized to replace the commercial polyester with the modified PGA in the preparation of nanoparticles [15,53,54,55,56,57,58,59]. In this study, PGA was modified with CH and VE as they have been shown to improve the stability of lipid-based nanoparticles [37,38]. The modification of PGA with CH and VE can alter the property of PGA possessing polymer–lipid hybrid characteristics, which can facilitate LPNP formation. Furthermore, the grafted PGAs were PEGylated with Mal-PEG to provide steric stabilization by the PEG chain and to functionalize the maleimide group with HYA-cys. Figure 1 illustrates the ATR-IR and ^1^H NMR spectra of the synthesized polymers. The typical peaks of the PGA main functional group were observed in the ATR-IR spectrum (Figure 1A) and were in agreement with previous reports [49,58]. Similarly, these peaks appeared in the spectra of PGA-CH, PGA-VE, and their Mal-PEG derivatives. The O-H stretching peaks of PGA-CH and PGA-VE over the range of 3500–3400 cm^−1^ were substantially diminished, suggesting the successful substitution of the hydroxyl groups of PGA by CH and VE. Other characteristics of CH and VE were detected, such as C=C stretching at 1680–1652 cm^−1^ and C-H stretching at 2960–2840 cm^−1^. In addition, the appearance of new C=O stretching at 1700 cm^−1^, C=O bending at 842 cm^−1^, and C-N stretching at 1342 cm^−1^ was attributed to the characteristics of the maleimide group of the Mal-PEG derivative polymers. The new absorption peaks at 2890 and 1109 cm^−1^ were assigned to the C-H and C-O-C stretching of the ethylene glycol unit of PEG. In the ^1^H NMR spectra (Figure 1B), the methylene protons of adipic units of all polymers were consistent with those of the previous reports [49,58]. The methyl (CH_3_) protons of CH appeared over the range of 0.75–1.08 ppm, whereas those of VE occurred in the range of 0.85–1.50 ppm and 2.02 ppm. The methine proton at 5.31 ppm indicated 1,2,3-tri-substituted glycerol repeating units [58], and this peak increased after CH and VE grafting. The new CH_2_ protons of the ethylene glycol unit of PEG occurred at 3.65 ppm. The M_n_ and M_w_/M_n_ of PGA were 6264 g/mol and 3.06, respectively (Table 1). When grafted with CHO and TOC, the M_n_ and M_w_/M_n_ values increased considerably due to a change in polymer architecture from linear to branch. However, the PEGylation of PGA-CH and PGA-VE slightly decreased these values because it altered the hydrophobicity of the polymers, resulting in a change in the solubility and in solvated volume in the GPC eluent. Taken together, these results provide clear evidence that the modifications of PGA with CH, VE, and Mal-PEG were successfully performed.

### 3.3. Characteristics of CME-Loaded LPNPs with and without HYA Conjugation

Recently, LPNPs have been introduced for applications in drug delivery because this system combines the advantages of lipid- and polymeric-based nanoparticles, which mainly comprise drugs, polymers, lipids, and stabilizers [35,36]. This study aimed to investigate the use of PGA-CH and PGA-VE for the preparation of LPNPs. In addition, Mal-PEG-PGA-CH and Mal-PEG-PGA-VE were employed in the formulations to mimic lipid-PEG and to functionalize the LPNPs with HYA-cys, a targeting ligand for CD44 receptors. The formulation of CME-loaded LPNPs was preliminarily optimized using PLGA as a polymeric component, which was commonly used to prepare LPNPs [36]. In the formulation, 25% weight of PC, based on the solid content of the organic phase, was used as a surfactant stabilizer during w/o/w emulsification and subsequently upon evaporation of the oil phase. PVA acted as a surfactant in the external water phase. The composition and characteristics of the LPNPs are shown in Table 2. It was found that the CME-PLGA LPNPs had a particle size of approximately 250 nm, a PDI value of 0.246 ± 0.039, a % DL of 1.66 ± 0.27%, and a %EE of 31.1 ± 6.8%, with a yield value higher than 78%. The ZP value was close to zero. Substituting PLGA with PGA-CH and PGA-VE dramatically affected the particle size and PDI of the CME-loaded LPNPs. Compared to the CME-PLGA LPNPs, the CME-CH LPNPs were approximately double in size and had a broad PDI value. The particle size and PDI of the CME-VE LPNPs increased by four- and two-fold, respectively. However, the surface charge of the CME-CH and CME-VE LPNPs (−5.8 ± 1.0 and −4.5 ± 1.8 mV, respectively) was slightly lower than that of the CME-PLGA LPNPs. The yield, DL, and EE were comparable to or slightly higher than those of the PLGA LPNPs. The bigger and broader size of the CME-CH and CME-VE LPNPs may result from the steric hindrance of polymer branching architectures and imbalanced hydrophobic/hydrophilic properties. Upon LPNP formation, the grafted CH and VE of PGA-CH and PGA-VE may interrupt the rearrangement of the lipids and polymers during the nanoparticle formation. Although the molecular weight of PLGA used in this study was higher than that of PGA-CH and PGA-VE, the hydrophobicity of PGA-CH and PGA-VE was greater. In general, PLGA, at the equal molecular weight and ratio of glycolide and lactide used in this study, has a water contact angle of 75°–98° [60,61]. PGA-CH and PGA-VE possessed considerably higher water contact angle values of 104° and 113°, respectively, suggesting much greater hydrophobicity of the polymers. The previous study found that an insufficient amount of PC led to increases in particle size and size distribution [37]. Therefore, the substitution of PLGA with PGA-CH and PGA-VE, even at an equal amount, may alter the optimal ratio of PC and the polymer and cause coalescence of oil droplets and low stability of emulsions upon particle formation, resulting in a bigger size and broader size distribution of LPNPs.

The PGA-CH- and PGA-VE-based formulations for the encapsulation of CME were further optimized by replacing a part of PGA-CH or PGA-VE with Mal-PEG-PGA-CH or Mal-PEG-PGA-VE, respectively, at 25 and 50% of the grafted polymer weight. The replacement with Mal-PEG-containing PGA polymers at 25 and 50% weight significantly decreased the particle size of the LPNPs by 2.2–3.3-fold (220–292 nm) and 2.5–4.5-fold (196–213 nm), respectively. In addition, the PDI values of the Mal-PEG-containing LPNPs were reduced by 1.2–1.9-fold and 1.7–2.1-fold, respectively. Considering the differences in the %weight of the Mal-PEG-containing grafted polymers, the LPNPs with 50% weight of the Mal-PEG-grafted polymers were smaller and had lower PDI values than those with 25% weight. This trend was observed for both the PGA-CH and the PGA-VE series. The other characteristics of the Mal-PEG-containing LPNPs were comparable with those of the PEG-free LPNPs. The presence of Mal-PEG in the formulations substantially assisted the formation of the LPNPs by sterically stabilizing the oil droplets and preventing coalescence upon particle solidification [36].

Comparing the PGA-CH and PGA-VE series, the PGA-CH-based formulations provided smaller sizes and lower PDI values than the PGA-VE-based LPNPs. CH generally functions as a stabilizer in liposomes and lipid-based nanoparticles [20]. In this study, the CH of PGA-CH and Mal-PEG-PGA-CH, rather than VE of PGA-VE and Mal-PEG-PGA-VE, may also act as a stabilizer upon oil droplet emulsification, thus preventing the coalescence of droplets and enhancing the stability upon the formation of LPNPs. From these results, the 50PEG/CH and 50PEG/VE formulations were employed for further study.

**Figure 2:**
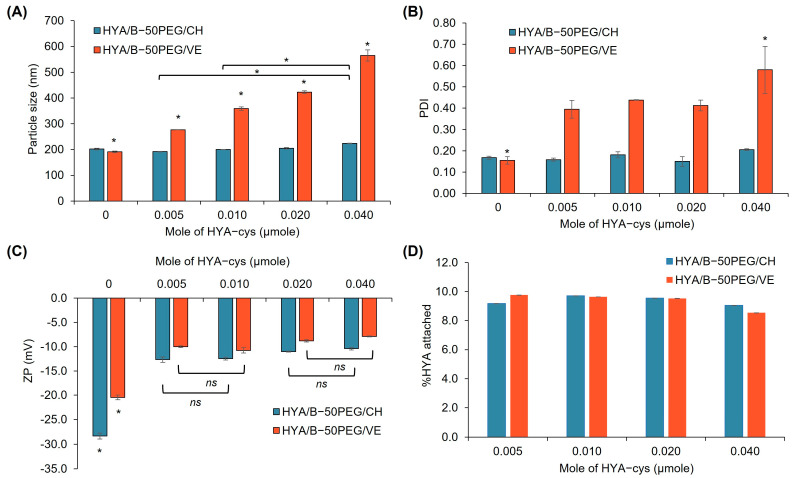
Particle size ((**A**), nm), polydispersity index ((**B**), PDI), zeta potential ((**C**), ZP, mV), and percentage of attached HYA (**D**) of blank 50PEG/CH and 50PEG/VE LPNPs before and after conjugation with HYA-cys at various moles of HYA. * Statistically significant difference when comparing among various moles of added HYA-cys; *^ns^* insignificant difference when comparing both amounts of added HYA-cys (*n* = 3).

To optimize HYA-cys conjugation on the surface of the LPNPs, the effect of various amounts of HYA-cys on the properties of the blank LPNPs was investigated. Previous studies demonstrated that different molecular sizes of HYA affected the cellular uptake and systemic exposure of the nanoparticles [62,63,64,65]. Although the high-MW HYAs showed higher uptake than the low-MW ones, the large-sized HYAs had higher blood clearance and lower exposure than the small-sized HYAs. Therefore, 8000–15,000 g/mol HYA was chosen for the LPNP surface conjugation. The optimization results are illustrated in Figure 2. In the case of PGA-CH LPNPs (HYA/B-50PEG/CH), various moles of HYA-cys did not affect the particle size and PDI of the LPNPs. Unlike the HYA/B-50PEG/CH LPNPs, the increasing amount of HYA-cys conjugation significantly enlarged the particle size and broadened the size distribution of the HYA/B-50PEG/VE LPNPs, suggesting the instability of the LPNPs. The highest amount of HYA-cys increased the particle size and PDI values by 2.9 (565 ± 22 nm) and 3.7 (0.580 ± 0.110) times, respectively, compared to the unconjugated LPNPs. After conjugation with HYA-cys, the surface charge of the HYA/B-50PEG/CH and HYA/B-50PEG/VE LPNPs changed to a significantly lower negative value (–10.4 to –12.7 mV and –7.9 to –10.8 mV, respectively). However, an increase in the amount of HYA-cys slightly reduced the negative surface charge but did not increase the amount of HYA conjugation on the surface of the LPNPs. From Ellman’s analysis, 8.54–9.76% of HYA was decorated on the surface of all LPNPs. The conjugation of HYA-cys on the surface of the LPNPs probably reached the plateau probably because HYA with the MW of 8000–15,000 g/mol may exert steric hindrance during the reaction. In addition to the effect of HYA size, the size of the particles and the surface density of HYA had impacts on the in vitro uptake and in vivo clearance of the particles [64]. The smaller-sized NPs entered the cells faster and higher than the larger particles, while high-density conjugated HYA had faster clearance from the blood. The optimum amount of HYA on the particle surface is important, and further pharmacokinetic study of the HYA-conjugated LPNPs may be needed. Hence, 0.005 µmole of HYA-cys was utilized to further decorate the 50PEG/CH and 50PEG/VE LPNPs.

**Figure 3:**
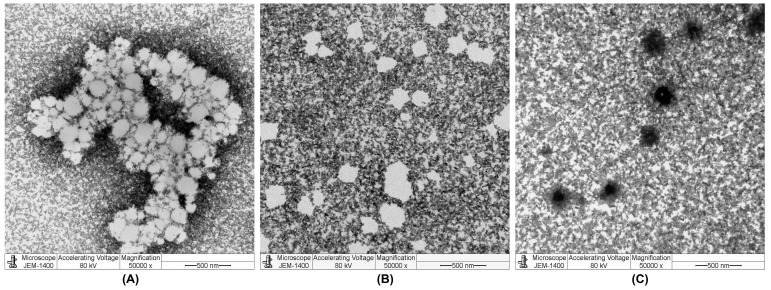
Examples of TEM images of blank 50PEG/CH LPNPs (**A**), 50PEG/VE LPNPs (**B**), and HYA/50PEG/CH LPNPs (**C**).

The TEM images of the blank LPNPs before and after HYA conjugation are shown in Figure 3. The images revealed that the blank 50PEG/CH LPNPs had a more spherical shape but were more aggregated than the 50PEG/VE LPNPs. After coating with HYA, the HYA/50PEG/CH LPNPs showed a more spherical shape and less aggregation. The HYA/50PEG/CH LPNPs were covered by uranyl acetate, as can be seen by the black surface, suggesting that the coating with HYA changed the surface properties of the LPNPs.

**Figure 4:**
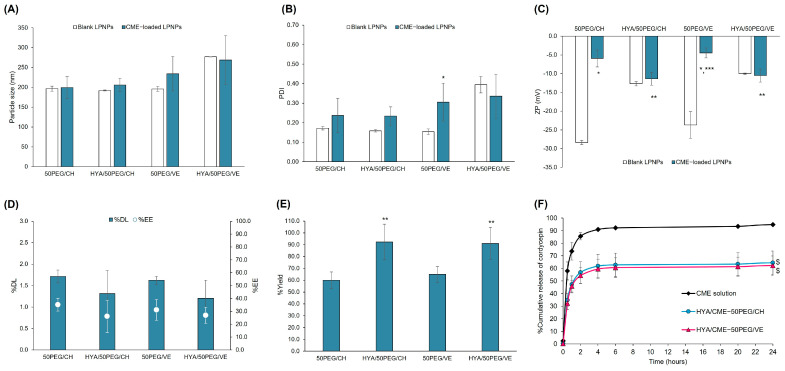
Characteristics (particle size ((**A**), nm), polydispersity index ((**B**), PDI), zeta potential ((**C**), ZP, mV), %drug loading ((**D**), %DL), %entrapment efficiency ((**E**), %EE), and release profiles (**F**)) of blank and CME-loaded 50PEG/CH and 50PEG/VE LPNPs with and without HYA conjugation. * Statistically significant difference when compared with the blank LPNPs; ** statistically significant difference when compared with the unconjugated LPNPs; *** statistically significant difference when comparing the PGA-CH and PGA-VE series at an equal amount of Mal-PEG used in the formulation; ^$^ statistically significant difference when compared with the CME solution (*n* ≥ 3).

The characteristics of the 50PEG/CH and 50PEG/VE LPNPs with and without HYA decoration are illustrated in Figure 4. The encapsulation of CME and the conjugation of HYA did not affect the particle size of the LPNPs compared to the blank and unconjugated LPNPs, respectively (*p* > 0.05). After CME encapsulation, the PDI values of most LPNPs slightly increased, except for the HYA/50PEG/VE LPNPs, whose values minimally decreased. However, the HYA conjugation did not affect the PDI values of the CME-loaded LPNPs. The CME significantly decreased the negative zeta potential of the unconjugated 50PEG/CH and 50PEG/VE LPNPs from –28.3 to –5.9 mV and –23.7 to –4.5 mV, respectively, suggesting that either CME or some the extract components may be adsorbed on the surface of the LPNPs. However, the zeta potential of the HYA/50PEG/CH and HYA/50PEG/VE LPNPs did not alter when loaded with CME. Compared to the unconjugated LPNPs, the zeta potential of the HYA-conjugated LPNPs became more negative (*p* < 0.05), indicating the successful conjugation of HYA on the surface of the LPNPs. The absolute zeta potential of the HYA-conjugated LPNPs was lower than 30 mV and may not be able to stabilize the LPNPs by electrostatic stabilization [66]. The conjugation of HYA and the presence of PEG on the surface of the LPNPs may possess steric stabilization for LPNP stability. However, the stability was not assessed in this study and remains a concern for future studies. Regarding loading capacity, the %DL of the CME-50PEG/CH and CM-50PEG/VE LPNPs ranged from 1.6% to 1.7%, and after surface decoration, the %DL was slightly reduced due to the dilution effect in the conjugation process. Nevertheless, %EE of HYA-conjugated and unconjugated LPNPs was comparable and was in the 26–35% range. The %yield of the HYA-conjugated LPNPs increased after conjugation to more than 90%. The release of CME was studied based on cordycepin, as shown in Figure 4F. It was found that the release of cordycepin from the HYA/50PEG/CH and HYA/50PEG/VE LPNPs was approximately 60% over 48 h; however, the CME solution released significantly faster and higher than the LPNPs. The release of cordycepin from the LPNPs agreed with that from liposomes [26] and gelatin nanoparticles [31]. The fast release of cordycepin within 48 h was due to its high water solubility. These results, together with the chemical characterizations, suggest that the fabrication of HYA-conjugated CME-loaded LPNPs using PGA-CH/Mal-PEG-PGA-CH and PGA-VE/Mal-PEG-PGA/VE polymers was successful.

### 3.4. Cytotoxicity of CME-Loaded LPNPs

**Figure 5:**
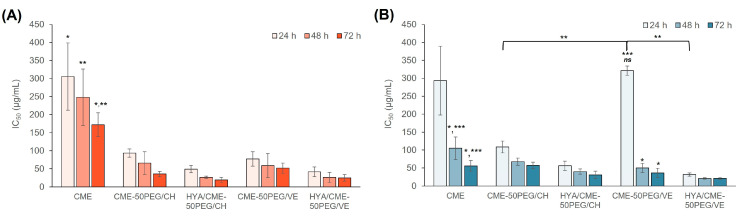
IC_50_ values of MDA-MB-231 (**A**) and MCF-7 (**B**) cells after treatment with CME solution, CME-50PEG/CH, CME-50PEG/VE, HYA/CME-50PEG/CH, and HYA/CME-50PEG/VE LPNPs for 24, 48, and 72 h (*n* = 3). * Statistically significant difference when compared with the value at 24 h; ** statistically significant difference when compared with the LPNP formulations; *** statistically significant difference when compared with the value of MDA-MB-231 cells; *^ns^* insignificant difference when compared with the CME solution.

The toxicity of the unconjugated and HYA-conjugated LPNPs was evaluated in MDA-MB-231 and MCF-7 breast cancer cells by an MTT assay. The CME solution was also tested for comparison. The cells were treated with CME-loaded LPNPs and CME solution for 24, 48, and 72 h. The IC_50_ values are illustrated in Figure 5. At 24 h, the CME solution had IC_50_ values of 305.6 ± 93.2 and 293.4 ± 95.9 µg/mL against MDA-MB-231 and MCF-7 cells, respectively. These values gradually decreased to 172.0 ± 33.0 and 55.6 ± 15.5 µg/mL, respectively, when the exposure time was extended to 72 h, suggesting a time-dependent anticancer effect. CME was significantly more toxic to MCF-7 cells than MDA-MB-231 cells, especially at 48 and 72 h of incubation. The toxicity of pure cordycepin in MDA-MB-231 and MCF-7 cells was further examined for 48 h. The results revealed that cordycepin had a higher IC_50_ value in MDA-MB-231 cells (61.7 µg/mL) than in MCF-7 cells (25.0 µg/mL). However, CME had approximately 4.0–4.2-fold higher IC_50_ values compared to cordycepin. Although CME contained a 10-fold smaller amount of cordycepin, the IC_50_ value was only four times lower. This result suggests that other components in the extract, which possessed an anticancer effect, such as ergosterol derivatives, ergothioneine, γ-butyric acid, or lovastatin [2,51,52], may additionally or synergistically exert the anticancer activity against these cell lines.

In the case of the CME-loaded LPNPs, all LPNPs had one to nine times lower IC_50_ values than the CME solution in both cell lines, suggesting greater toxicity toward the cells when the CME was encapsulated in the LPNPs. In addition, these values further decreased with increasing exposure time, implying a time-dependent anticancer effect of CME-loaded LPNPs. The unconjugated LPNPs caused 3.2–4.8- and 0.9–2.7-fold more toxicity toward MDA-MB-231 and MCF-7 cells, respectively, compared to the CME solution, except for CME-50PEG/VE at 24 h and CME-50PEG/CH at 72 h toward MCF-7 cells. Although the release of cordycepin almost reached a plateau after 20 h, the anticancer effect of the CME-loaded LPNPs was sustained until 72 h. This may be attributed to other bioactive compounds entrapped in the LPNPs and slowly released from the particles. In addition, CME may affect the proliferation or growth phase of the cells; thus, a prolonged time to cause cell death is required [67]. The surface conjugation of the LPNPs with HYA enhanced the toxicity toward both cells by 6.2–9.5 and 1.8–9.2 times, respectively, compared to the CME solution and by 1.9–2.5 and 1.7–10.1 times, compared to the unconjugated LPNPs. The existence of CD44 receptors on the cell surfaces of both cells caused the enhanced effect of the HYA-conjugated LPNPs. According to the analysis of the gene expression (Table S2, Supporting Information), the CD44 receptor gene was expressed in both cell lines. This led to the enhanced anticancer effect of the HYA-conjugated CME-loaded LPNPs compared to the unconjugated formulations.

Comparing the PGA-CH and PGA-VE series, the 50PEG/VE LPNPs had a more pronounced anticancer effect than the 50PEG/CH LPNPs on MCF-7 cells, except for CME-50PEG/VE at 24 h; however, they had a comparable effect on MDA-MB-231 cells. The enhanced effect of the 50PEG/VE LPNPs compared to the 50PEG/CH LPNPs was associated with the presence of VE in the LPNPs. Since VE exerts an anticancer effect in adjuvant cancer therapy [68], the presence of VE in LPNPs may additionally enhance toxicity toward both cells. These results suggest that the fabricated unconjugated and HYA-conjugated LPNPs successfully enhanced the anticancer effect of CME.

### 3.5. Cellular Uptake

**Figure 6:**
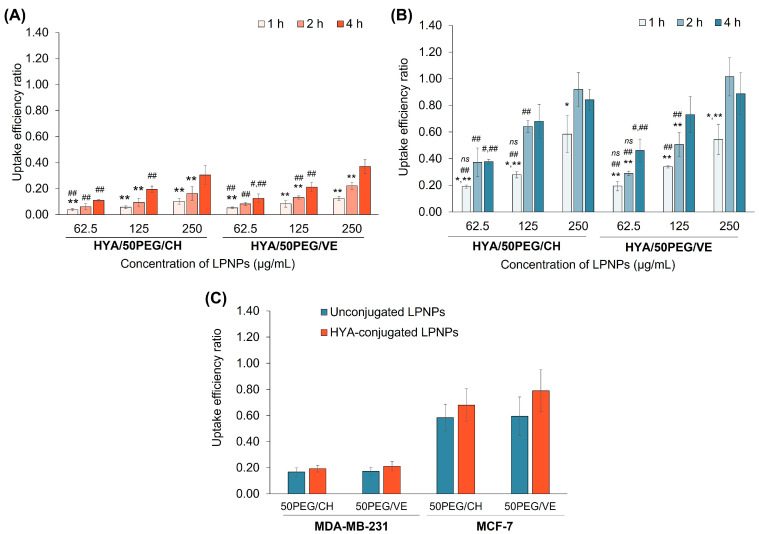
Uptake efficiency ratio of MDA-MB-231 (**A**) and MCF-7 (**B**) cells after incubation with various concentrations of coumarin-labeled HYA/50PEG/CH and HYA/50PEG/VE LPNPs for 1, 2, and 4 h (*n* = 3). (**C**) Uptake efficiency ratio of MDA-MB-231 and MCF-7 cells after incubation with 125 µg/mL coumarin-labeled unconjugated and HYA-conjugated LPNPs for 4 h. *,** Statistically significant difference when comparing the uptake efficiency ratio at any time with the values at 2 and 4 h, respectively; ^#,##^ statistically significant difference when comparing the values of LPNPs at the designated concentration with 125 and 250 µg/mL, respectively; *^ns^* insignificant difference when comparing the uptake efficiency in MCF-7 cells with that in MDA-MB-231 cells after treatment with the same concentration of LPNPs.

The cellular uptake of HYA-conjugated LPNPs was investigated in both cell lines using flow cytometry. The results are expressed as the uptake efficiency ratio, as shown in Figure 6. The uptake efficiency of HYA-conjugated LPNPs was time- and concentration-dependent in MDA-MB-231 cells. In MCF-7 cells, the uptake efficiency increased with time and concentration; however, it reached a plateau after 2–4 h of incubation. Comparing both cell lines, the particle uptake by MCF-7 cells was higher than that by MDA-MB-231 cells. It was postulated that the fabricated LPNPs may enter the cells through CD44 receptors. HYA has been employed as a targeting ligand and extensively decorated on the nanoparticle surface, which can bind to the CD44 receptors of cancer cells [21,22,69]. Thus, the expression of the CD44 receptors was determined by qPCR. It was found that the gene expression of the CD44 receptors in MCF-7 cells was two times higher than that of MDA-MB-231 cells (Table S2). Based on the results, the uptake of the HYA-conjugated LPNPs depended on the exposure time and particle concentration and was directly related to the expression of the CD44 receptor gene. In addition, the uptake of HYA-conjugated LPNPs was studied by comparison with the unconjugated formulations. As shown in Figure 6C, the HYA-conjugated LPNPs exhibited 1.2–1.3 times higher uptake efficiency than the unconjugated LPNPs (uptake efficiency ratios in the ranges of 0.19–0.79 and 0.17–0.60, respectively). Therefore, the HYA-conjugated LPNPs entered the cells through CD44 receptor-mediated endocytosis. Comparing the PGA-CH and PGA-VE series, the uptake of the HYA/50PEG/VE LPNPs was marginally higher than that of the HYA/50PEG/CH LPNPs.

**Figure 7:**
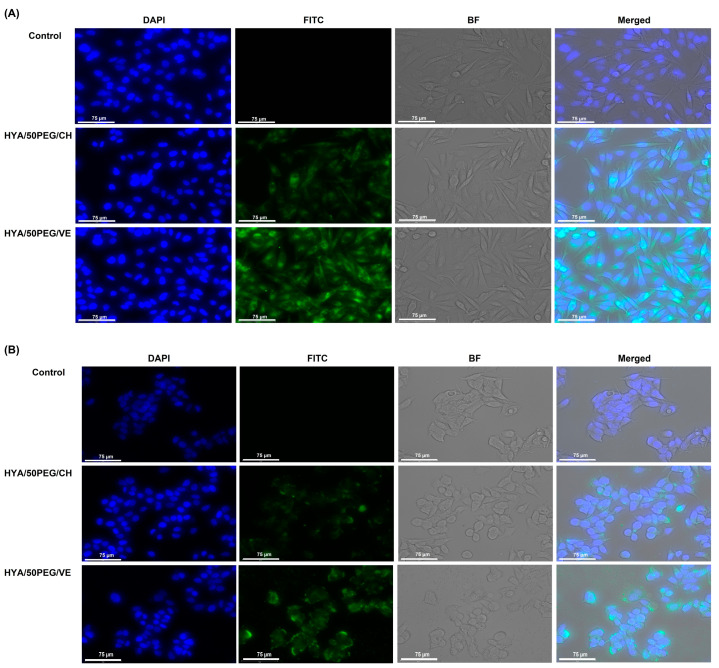
Cellular images of MDA-MB-231 (**A**) and MCF-7 (**B**) cells after incubation with 125 µg/mL of coumarin-labeled HYA/50PEG/CH and HYA/50PEG/VE LPNPs for 2 h. A scale bar indicates 75 µm. The cells were captured in four channels; DAPI, FITC, BF, and merged images illustrate Hoechst 33342-staining nuclei, coumarin-6-labeled LPNPs, bright field of the cells, and overlaying of DAPI, FITC, and BF channels, respectively.

The uptake of the LPNPs was visualized by a cell imaging system, as shown in Figure 7. The cells were exposed to 125 µg/mL of coumarin-6-labled HYA-conjugated LPNPs for 2 h. The green and blue fluorescence indicated the location of the LPNPs and nuclei, respectively. In the control group, both cells had no autofluorescence in the FITC channel. The green spots of both cells in the FITC and merged channels intracellularly localized around the nucleus of the cells, suggesting the localization of LPNPs in the cytoplasm after entering the cells. The cells treated with the HYA/50PEG/VE LPNPs exhibited more intensified green fluorescence than those treated with the HYA/50PEG/CH LPNPs; this is consistent with the uptake efficiency results.

These results confirmed the successful delivery of CME by HYA-conjugated PGA-based LPNPs through CD44 receptors in MCF-7 and MDA-MB-231 breast cancer cells. Combining the uptake and cytotoxicity results, the HYA/50PEG/VE LPNPs could enter the cells higher than the HYA/50PEG/CH LPNPs, resulting in the greater cytotoxicity. The developed HYA-conjugated LPNPs could deliver the extract more effectively to MCF-7 cells than MDA-MB-231 cells due to the combined effects of a higher fraction of LPNPs taken up by the cells and more sensitivity to CME.

## 4. Conclusions

This study demonstrates the first successful delivery of CME to tumor cells by HYA-conjugated LPNPs targeting CD44 receptors. The developed LPNPs clearly enhanced the anticancer activity of CME against MCF-7 and MDA-MB-231 breast cancer cells through improved cellular uptake. In this work, PGA-CH- and PGA-VE-based polymers were, for the first time, successfully used as polymeric components of LPNP complex systems. These materials combine the properties of PGA as a polymer core and CH and VE as grafted lipophilic moieties. Both the PGA-CH and the PGA-VE series of LPNPs exhibited the comparable toxicity and cellular uptake. However, the stability of the LPNPs was not assessed and their stability is of concern. Further study is required for the future use of the developed LPNPs for CME delivery. The developed LPNPs showed clear potential for the targeted delivery of herbal extracts for cancer treatment in in vitro tests, with a promising opportunity for translation in in vivo experiments. Nevertheless, the in vivo study may be required to demonstrate the selectivity and efficacy of the developed LPNPs toward tumors.

## Figures and Tables

**Table 1:** Number-average molecular weight (M_n_) and molecular weight distribution (M_w_/M_n_) of the synthesized polymers.

Polymers	M_n_ (g/mol)	M_w_/M_n_
PGA	6300	3.1
PGA-CH	10,600	5.4
Mal-PEG-PGA-CH	9900	4.8
PGA-VE	10,100	6.4
Mal-PEG-PGA-VE	9900	4.3

**Table 2:** Compositions in the organic phase and characteristics of CME-loaded LPNPs without HYA coating.

Series	Formulation Code	Weight (mg) of Components Used in Organic Phase				Mean (SD)					
		PLGA	PGA-CH/ PGA-VE	Mal-PEG-PGA-CH/ Mal-PEG-PGA-VE	PC	Size (nm)	PDI	ZP (mV)	%Yield	%DL	%EE
PLGA	CME-PLGA	18.75	-	-	6.25	251 (49)	0.246 (0.039)	–2.2 (1.0)	78.0 (4.8)	1.66 (0.27)	31.1 (6.8)
PGA-CH	CME-CH	-	18.75	-	6.25	491 *** (290)	0.428 (0.201)	–5.8 * (0.4)	81.0 (8.3)	1.64 (0.13)	31.6 (2.9)
	CME-25PEG/CH	-	14.06	4.69	6.25	220 (21)	0.227 (0.032)	–6.6 * (1.2)	80.7 (17.7)	1.49 (0.35)	27.8 (3.9)
	CME-50PEG/CH	-	9.38	9.38	6.25	196 (7)	0.201 (0.021)	–5.4 * (1.0)	85.8 (5.1)	1.71 (0.15)	35.2 (4.9)
PGA-VE	CME-VE	-	18.75	-	6.25	969 * (397)	0.555 * (0.351)	–4.5 (1.8)	73.8 (0.7)	1.72 (0.32)	31.0 (9.2)
	CME-25PEG/VE	-	14.06	4.69	6.25	292 ** (17)	0.450 (0.049)	–5.9 * (1.8)	75.4 (5.0)	1.56 (0.22)	28.8 (9.0)
	CME-50PEG/VE	-	9.38	9.38	6.25	213 ** (28)	0.312 (0.137)	–4.0 (2.0)	78.6 (7.6)	1.62 (0.09)	31.1 (8.1)

* Statistically significant difference when comparing PGA-based formulations with PLGA formulations; ** statistically significant difference when comparing PEG-PGA-based formulations with PEG-free formulations containing similar grafting moieties; *** statistically significant difference when comparing PGA-CH-based LPNPs with PGA-VE-based formulations containing equal ratios of polymers.

## Data Availability

The data presented in this study are available on request from the corresponding author.

## Supplementary Materials

The following supporting information can be downloaded at: https://www.mdpi.com/article/10.3390/pharmaceutics15061771/s1, Figure S1: 1H NMR spectrum of HYA-cys; Figure S2: HPLC chromatograms of cordycepin standard (A) and CME (B); Table S1: Extraction conditions, percentages of cordycepin and adenosine contents, and yield of CM extracted by ultrasonic-assisted and conventional methods; Table S2: C_t_, ∆C_t_, and 2^−∆Ct^ of CD44 and β-actin genes of MDA-MB-231 and MCF-7 cells. References [70,71] are cited in the supplementary materials.
