# Supplementary material for: CD44-Targeted Lipid Polymer Hybrid Nanoparticles Enhance Anti-Breast Cancer Effect of Cordyceps militaris Extracts
Source: Pharmaceutics. 2023 Jun 20;15(6):1771. doi: 10.3390/pharmaceutics15061771 (PMC10302493; doi:10.3390/pharmaceutics15061771)
Supplement: Supplementary file 1 [file pharmaceutics-15-01771-s001.zip › pharmaceutics-2438673-supplementary.pdf]

Supporting information

CD44-Targeted Lipid Polymer Hybrid Nanoparticles Enhance Anti-Breast Cancer Effect of *Cordyceps militaris* Extracts

1. Thiolation of HYA

Thiolation of HYA was performed according to the published method [1] by conjugation of HYA with cys using EDC/NHS as coupling agents. In brief, 300 mg HYA was dissolved in 30 mL deionized water with subsequent additions of EDC (17.54 mg, 0.11 mmol) and NHS (13.00 mg, 0.11 mmol). The pH of the solution was adjusted to 5.0 with 0.1 M HCl, and the solution was stirred at room temperature for 30 min. Cys (13.69 mg, 0.11 mmol) was added, and the reaction was conducted at room temperature for another 4 hours. The mixture was dialyzed using a dialysis bag (MWCO 3.5 kDa) against diluted HCl (pH 5.0), 0.9% NaCl in dilute HCl, dilute HCl (pH 5.0), and finally deionized water. The resultant was lyophilized for 48 h and the dried HYA-cys was kept at 4°C for further use. The ^{1}H NMR spectrum of HYA-cys in D2O is shown in **Figure S1**.

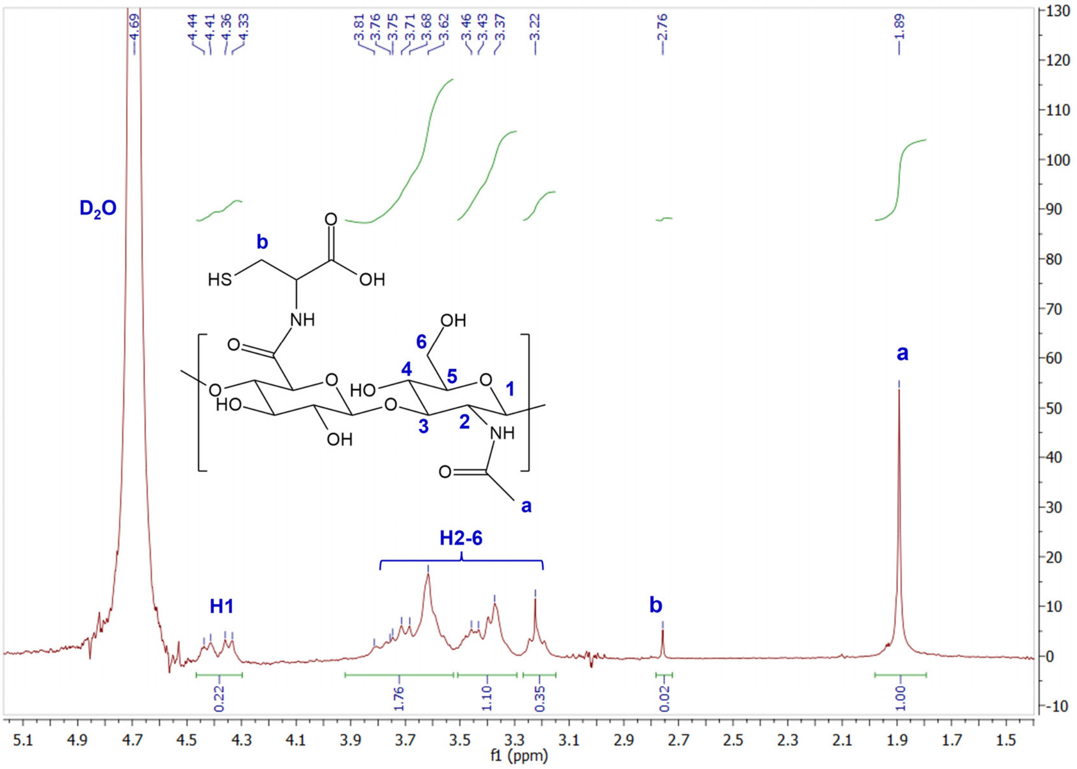

Figure S1. ^{1}H NMR spectrum of HYA-cys.

2. HPLC Chromatograms of CME

The HPLC chromatogram of CME in comparison with that of the cordycepin standard is illustrated in **Figure S2**.

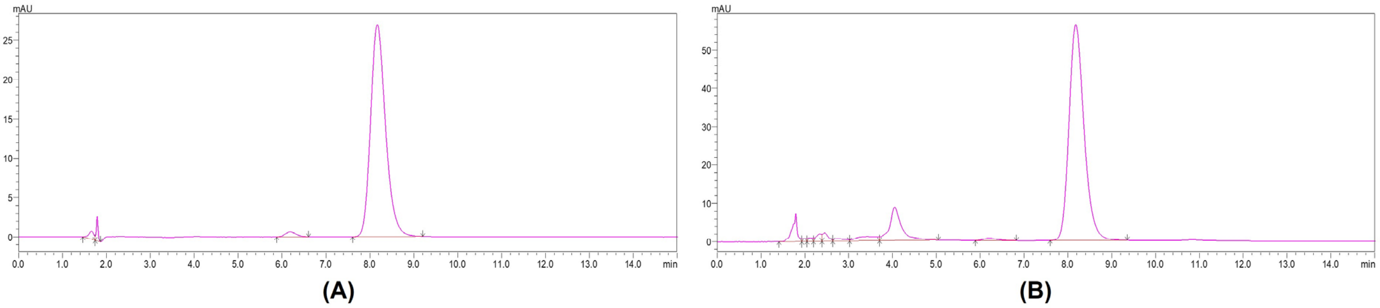

Figure S2. HPLC chromatograms of the cordycepin standard (A) and CME (B).

2. Extraction of CM by Conventional Method

One gram of pulverized fruiting body was wet and percolated with 100 mL of 50% v/v ethanol. The sample was shaken at 150 rpm, 60°C for 24 h using an incubator shaker. The sample was filtered through Whatman No. 1 filter paper, and the filtrate was collected for drying. The extraction of residual mass was repeated twice. All filtrates were combined and removed by a rotary evaporator at 45 °C. The crude extract was dried using a Christ Alpha 1-4 lyophilizer (Martin Christ Gefriertrocknungsanlagen GmbH, Göttingen, Germany) for 48 h. The dried extract was kept in a desiccator and light-protected until further use. The comparative conditions and preliminary results of CM extraction are summarized in **Table S1**.

Table S1. Extraction conditions, percentages of cordycepin and adenosine contents, and yield of CM extracted by ultrasonic-assisted and conventional methods

Extraction methods	Extraction condition			Percentages		
	Volume of solvent (ml)	Temperature (°C)	Time (h)	Cordycepin content	Adenosine content	Yield
Ultrasonic-assisted	20	60	1	10.55±0.01	0.046±0.002	9.89
Conventional	100	60	24	11.89±0.88	0.066±0.015	7.48

3. Determination of CD44 Receptor Gene Expression

The expression of CD44 receptors in MDA-MB-231 and MCF-7 cells was examined by quantitative polymerase chain reaction (qPCR). The cells were seeded in a 6-well plate at a density of 5×10^5 cells/well and cultured in DMEM supplemented with 10 % FBS and 1 % penicillin/streptomycin in a 5 % CO_2 humidified incubator at 37°C. Until the confluency reached 80–90 %, the cells were trypsinized. GenUP™ Total RNA Kit (Biotechrabbit GmbH, Berlin, Germany) was employed to extract RNA, which was then converted to cDNA by RevertUP™ II reverse transcriptase (Biotechrabbit GmbH, Berlin, Germany). The detection of gene expression by qPCR was performed using KAPA SYBR® FAST qPCR master mix (2X) kit. In this study, β-actin was utilized as a reference housekeeping gene, and the primer genes were as follows; β-actin forward: 5′-GATTCCTATGTGGGCGACGAG-3′ and reverse: 5′-CCATCTCTTGCTCGAAGTCC-3′ [2]; CD44 forward: 5′-CCAGAAGGAACAGTGGTTTGGC-3′ and reverse: 5′-ACTGTCCTCTGGGCTTGGTGTT-3′ [3].

The gene expression is expressed as C_t, ΔC_t, and $2^{-\Delta Ct}$ to the reference gene, as shown in **Table S2**. The $2^{-\Delta Ct}$ values indicate the expression amount of the test gene (CD44) compared to reference gene (β-actin). It was found that MCF-7 cells expressed the CD44 receptor gene more than MDA-MB-231 cells by approximately 2 folds.

Table S2. C_t, ΔC_t, and $2^{-\Delta Ct}$ of CD44 and β-actin genes of MDA-MB-231 and MCF-7 cells

Cells	C_t (CD44)	C_t (β-actin)	ΔC_t [a]	$2^{-\Delta Ct}$
MDA-MB-231	26.74	21.11	5.63	0.020
MCF-7	24.94	20.27	4.67	0.039

[a] $\Delta C_t = C_t(CD44)-C_t(\beta\text{-actin})$

References

1. Fan, Y.; Sahdev, P.; Ochyl, L.J.; J. Akerberg, J.; Moon, J.J. Cationic liposome–hyaluronic acid hybrid nanoparticles for intranasal vaccination with subunit antigens. *Journal of Controlled Release* **2015**, *208*, 121-129, doi:https://doi.org/10.1016/j.jconrel.2015.04.010.
2. Kendirgi, F.; Rexer, D.J.; Alcázar-Román, A.R.; Onishko, H.M.; Wente, S.R. Interaction between the shuttling mRNA export factor Gle1 and the nucleoporin hCG1: a conserved mechanism in the export of Hsp70 mRNA. *Mol. Biol. Cell* **2005**, *16*, 4304-4315, doi:10.1091/mbc.e04-11-0998.
3. Narvaez, C.J.; LaPorta, E.; Robilotto, S.; Liang, J.; Welsh, J. Inhibition of HAS2 and hyaluronic acid production by 1,25-dihydroxyvitamin D(3) in breast cancer. *Oncotarget* **2020**, *11*, 2889-2905, doi:10.18632/oncotarget.27587.
